# Healthy dietary patterns improve sexual function and incontinence symptoms: systematic review and meta-analysis of dietary patterns and dietary interventions

**DOI:** 10.3389/fnut.2025.1635909

**Published:** 2025-09-30

**Authors:** Daiwen Xing, Min Li, Yifei Zhong, Lin Liang, Huiqing Yao, Yaxin Liang, Yuhan Lyu, Yue Yu

**Affiliations:** ^1^Department of Gynecology and Obstetrics, National Center of Gerontology, Institute of Geriatric Medicine, Beijing Hospital, Chinese Academy of Medical Sciences, Beijing, China; ^2^Beijing Key Laboratory of Drug Clinical Risk and Personalized Medication Evaluation, Clinical Trial Center, National Center of Gerontology, Institute of Geriatric Medicine, Beijing Hospital, Chinese Academy of Medical Sciences, Beijing, China; ^3^The Key Laboratory of Geriatrics, National Center of Gerontology, Beijing Institute of Geriatrics, Institute of Geriatric Medicine, Beijing Hospital, Chinese Academy of Medical Sciences, Beijing, China; ^4^Chinese Academy of Medical Sciences and Peking Union Medical College, National Center of Gerontology, Institute of Geriatric Medicine, Beijing Hospital, Beijing, China

**Keywords:** pelvic floor dysfunction (PFD), mediterranean diet, DASH diet, anti-inflammatory diet, pro-inflammatory diet, sexual dysfunction, incontinence symptoms

## Abstract

**Background:**

Pelvic floor dysfunction (PFD) primarily including urinary incontinence, fecal incontinence, and sexual dysfunction, significantly impairs individuals’ quality of life. While healthy dietary patterns are considered potential modulators, a synthesized understanding of their impact is lacking.

**Objective:**

This systematic study aims to assess the effects of various healthy dietary patterns on PFD, providing a scientific basis for developing effective dietary intervention strategies in clinical practice.

**Methods:**

Following PRISMA guidelines, we systematically searched PubMed, Web of Sciences, and Embase databases. A total of 493 articles were identified across the five evidence-based dietary patterns: DASH, Mediterranean diet, hPDI, anti-inflammatory diet, and pro-inflammatory diet. After screening titles and abstracts, 196 articles were selected for full-text review, and 31 studies met the criteria. Of these, 14 studies provided sufficient quantitative data and were ultimately included in the meta-analysis. Statistical analyses, including odds ratios (OR) for cross-sectional studies and standardized mean differences (SMD) for prospective studies, were conducted using Review manager version 5.4.

**Results:**

This study included 10 prospective studies, 17 cross-sectional studies, and 4 randomized controlled trials (RCTs) that recruited diabetic patients, patients with urinary incontinence, and other populations from North America, Europe, Asia, and other regions. The findings showed that healthy dietary patterns significantly improved sexual dysfunction (cross-sectional studies: OR = 0.69, 95% CI [0.55, 0.85]; prospective studies: SMD = −0.6, 95% CI [−1.02, −0.17]) and incontinence symptoms (cross-sectional studies: OR = 0.77, 95% CI [0.68, 0.87]). Specially, the Mediterranean diet and anti-inflammatory dietary patterns were significantly associated with a reduced risk of sexual dysfunction. The DASH diet was effective in alleviating lower urinary tract symptoms and urgency urinary incontinence. Conversely, pro-inflammatory dietary patterns were significantly associated with an increased risk of urinary and fecal incontinence.

**Conclusion:**

Healthy, anti-inflammatory dietary patterns, particularly the Mediterranean diet, are associated with significant improvements in Pelvic floor dysfunction, particularly sexual dysfunction and incontinence symptoms. These findings support the integration of dietary counseling into the management of PFD, with a particular focus on patients with co-existing metabolic risk factors.

## 1 Introduction

Pelvic floor dysfunctions (PFD) is a common but complex syndrome that includes symptoms such as urinary incontinence, sexual dysfunction, anal incontinence, and pelvic organ prolapse. The presence of any single symptom may be defined as pelvic floor dysfunction. Additionally, sexual dysfunction, including decreased libido, dyspareunia, orgasmic dysfunction and erectile dysfunction, are common in both men and women.(1) Given that lifestyle modification is a first-line treatment, (2) understanding the pivotal role of diet is crucial (3, 4).

Healthy dietary patterns, as a non-pharmacological intervention, have been widely studied and shown to play a critical role in the prevention and management of various chronic diseases (5). Diets known to be anti-inflammatory, such as the Mediterranean diet, have been associated with improved sexual function, (6) while reducing salt intake can improve overactive bladder symptoms (7). Conversely, pro-inflammatory diets, often quantified by the Dietary Inflammatory Index (DII), are linked to an increased risk of UI (8, 9). These distinct dietary patterns, therefore, represent opposing ends of the inflammation spectrum, providing a strong rationale for their combined evaluation.

Despite these links, the evidence remains fragmented, often focusing on single nutrients or small-scale observational studies rather than the synergistic effects of whole dietary patterns. A large-scale, systematic synthesis of the evidence is currently lacking. Therefore, this study aims to integrate existing research, combining cross-sectional and prospective studies, to evaluate the correlation between different dietary patterns and PFD through meta-analysis. Additionally, it seeks to examine whether specific dietary patterns lead to different outcomes through randomized controlled trials (RCTs), proposing evidence-based intervention strategies for clinical practice.

## 2 Materials and methods

This systematic review adhered to the Preferred Reporting Items for Systematic Reviews and Meta-analyses (PRISMA) (10) guidelines and followed a pre-planned unpublished protocol that can be requested by contacting the corresponding author. This systematic review was registered in the International Prospective Register of Systematic Reviews (PROSPERO) under the number CRD42024616704.

### 2.1 Search strategy

**FIGURE 1:**
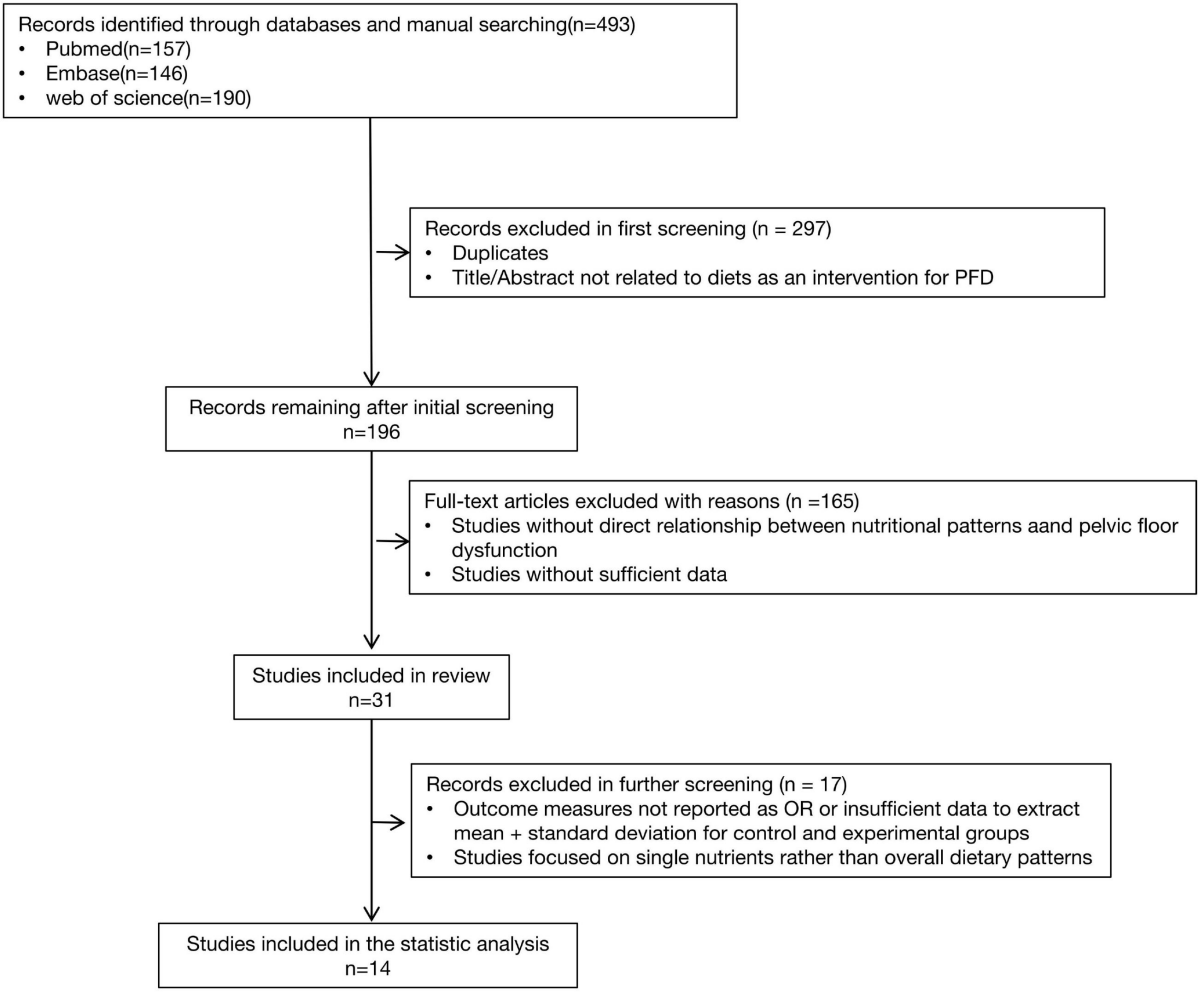
Flow diagram of systematic review.

This study evaluates the effects of the DASH diet, Mediterranean diet, WCRF/AICR diet, hPDI diet, AHEI-2010, and pro-inflammatory or anti-inflammatory diets on chronic pelvic floor dysfunction diseases (PFD). Two authors conducted literature searches in PubMed, Web of Sciences, and Embase for relevant publications up to August 2024. The systematic search employed both Medical Subject Headings (MeSH) and non-MeSH keywords, including the following terms in titles and abstracts: “DASH” OR “Diet, Sodium-Restricted” OR “Dietary Approaches to Stop Hypertension” OR “Mediterranean diet” OR “Diet, Mediterranean” OR “Healthful Plant-Based Diet Index” OR “Diet, Vegetarian” OR “hPDI” OR “pro-inflammatory diet” OR “Anti-Inflammatory Diet” OR “dietary inflammatory index” AND “pelvic floor dysfunction” OR “pelvic floor disorder” OR “pelvic organ prolapse” OR “urinary incontinence” OR “fecal incontinence” OR “bladder storage” OR “lower urinary tract symptoms” OR “sexual dysfunction” OR “chronic pelvic pain”. The subsequent literature screening process is shown in Figure 1.

### 2.2 Study selection

Study eligibility was defined according to the Participant, Intervention, Comparator,

Outcome, Study type (PICOS) framework (11). The authors reviewed the titles and abstracts of all identified articles, selecting studies that met the following inclusion criteria: (1) full text available; and (2) studies assessing the relationship between dietary patterns (in cohort studies) or dietary interventions (in trials) and relevant outcomes (e.g., sexual function scores, lower urinary tract symptom scores).

Both cross-sectional and prospective studies were included. As cross-sectional studies provide a broad overview of the population, while prospective studies offer evidence of causality. By combining both study types, this review compensates for the limitations of each individual design, offering more reliable evidence regarding the link between healthy dietary patterns and PFD.

### 2.3 Data extraction

Data extracted from each study included the following: reference (author, year), country, population details (clinical characteristics, sample size, age, and follow-up time), dietary assessment tools and their main relevant features, outcomes, and adjustments for potential confounders. When reporting multivariate models, the model containing the most appropriate set of potential confounders was selected.

### 2.4 Outcomes

For cross-sectional studies, the primary outcome was usually the odds ratio (OR). For prospective studies, the primary outcome was the mean ± standard deviation (SD).

### 2.5 Bias assessment

The risk of bias was evaluated using the Newcastle-Ottawa Scale (NOS) for non-randomized controlled trials (non-RCTs) (Table 1), and the Cochrane risk assessment tool was employed for RCTs (12). The NOS uses a scoring system with a maximum score of 9 points, where a score of 7–9 indicates a low risk of bias, and a score of 4–6 indicates a high risk of bias.

**TABLE 1:** NOS for risk of bias and quality assessment in cohort studies, case-control studies, and cross-sectional studies.

Cohort Studies									
	Selection				Comparability	Exposure			Total score
Author year	Representativeness of exposed cohort	Selection of the non exposed cohort	Ascertaiment of exposure	Demonstration that outcome of interest was not present at start of study	Comparability of cohorts on the basis of the design or analysis	Assessment of outcome	Was follow-up long enough for outcomes to occur	Adequacy of follow up of cohorts	
Matsuo et al. (13)	1	1	1	1	1	1	1	1	8
Matsuo et al. (7)	1	1	1	0	2	1	1	1	8
Loeb et al. (14)	1	1	1	0	2	0	1	1	7
**Case-control and cross -sectional studies**									
	**selection**				**Comparability**	**Exposure**			**Total score**
**Author year**	**Adequate case definition**	**Representativeness of the cases**	**Selection of controls**	**Definition of controls**	**Comparability of cases and controls on the basis of the design or analysis**	**Ascertainment of exposure**	**Same method of ascertainment for cases and controls**	**Non-Response rate**	
Li et al. (15)	1	1	1	1	2	1	1	0	8
Liao et al. (16)	1	1	1	1	2	1	1	0	8
Ruan et al. (17)	1	1	1	1	2	1	1	0	8
Zhang et al. (18)	1	1	1	1	2	1	1	0	8
Glugiano et al. (19)	1	1	1	1	2	1	1	1	9
Glugiano et al. (20)	1	1	1	1	2	1	1	1	9
Carto et al. (21)	1	1	1	1	2	1	1	0	8
Fantus et al. (22)	1	1	1	1	2	1	1	0	8

NOS, Newcastle-Ottawa scale. 1: 1 point; meets NOS criteria for high quality research. 0: 0 points; does not meet NOS criteria for high quality research.

Two reviewers independently assessed each study’s quality. Discrepancies were resolved through discussion and major disagreements were brought to a third reviewer to reach a consensus.

### 2.6 Meta-analysis

Only studies meeting the inclusion criteria were included in the meta-analysis. Statistical analyses were independently performed by two authors using Review Manager version 5.4 (The Cochrane Collaboration, Software Update, Oxford, London). Discrepancies in results were discussed and resolved by a third author. Continuous variables were compared using means and standard deviations, while dichotomous variables were compared using odds ratios (OR). Results are presented as OR or standard mean differences (SMD) with 95% confidence intervals. To standardize the effects of different dietary patterns on urinary incontinence and fecal incontinence, inverse transformation was applied to the pro-inflammatory diet data. The level of significance was set at *p* < 0.05. Heterogeneity was assessed using the Higgins I^2^ statistic. The results are presented in a forest plot.

## 3 Results

Figure 1 depicts the number of studies screened, evaluated for eligibility, and ultimately included in the review. While the strategy also included terms for the WCRF/AICR diet and the AHEI-2010, no studies evaluating these specific dietary patterns met our final inclusion criteria. Table 2 provides a summary of the main characteristics of the included studies. Among the 31 full-text articles reviewed, 14 met the criteria for statistical analysis, comprising 8 cross-sectional studies, 3 RCTs and 3 prospective studies. Nutrient intake was predominantly assessed using the NHANES dietary questionnaire or the Food Frequency Questionnaire (FFQ). For sexual function assessment, Fantus and Ruan used self-reported erectile function data from NHANES, while Giugliano, Esposito, and Maiorino et al. utilized the International Index of Erectile Function-5 (IIEF-5) for male sexual dysfunction and the Female Sexual Function Index (FSFI) for female sexual dysfunction. Loeb et al. employed the Expanded Prostate Cancer Index Composite (EPIC) to evaluate lower urinary tract symptoms and sexual dysfunction. For the assessment of urinary incontinence, Zhang and Liao used self-reported data, whereas Matsuo employed the Overactive Bladder Symptom Score (OABSS) questionnaire and the Core Lower Urinary Tract Symptoms Score (CLASS). For fecal incontinence, Li et al. used the Bowel Health Questionnaire (BHQ).

**TABLE 2:** Characteristics of included studies (*n* = 14).

Descriptive study (Cross-sectional study)								
Study	Symptom	Country	study period	Population *n* (% female)	Dietary pattern	Outcomes model (logistic regression, linear regression) OR Q5 vs. Q1:;95%CI	Observations	
Giugliano et al. (19)	sexual dysfunction	Italy	Not mentioned	Type 2 diabetes *n* = 555(0%)	Mediterranean diet	Prevalence of ED (IIEF-5 ≤ 21): 3rd tertile vs. 1st tertile:52.6% vs. 61.2%,*P* = 0.01	Models adjusted for age, BMI, waist circumference, WHR, physical activity, smoking status, hypertension, diabetic medication use, duration of diabetes, and total and high-density lipoprotein cholesterol, triglyceride, HbA1c and glucose concentrations	
Giugliano et al. (20)		Italy	Not mentioned	Type 2 diabetes *n* = 595(100%)	Mediterranean diet	Prevalence of FSD (FSFI ≤ 26): 3rd tertile vs. 1st tertile:49.1% vs. 57.6%, *P* = 0.01	–	
Fantus et al. (22)		U.S	2001–2004	*n* = 4027(0%)	Mediterranean diet	Multinomial logistic regression: OR: 0.749,*P* = 0.430	–	
Ruan et al. (17)		U.S.	2001–2004	*n* = 3693(0%)	PID	Sample-weighted logistic regression: OR Tertile3 vs. Tertile1: 1.51, 95% CI1.09–2.10	Models adjusted for age, race, ethnicity, education, physical activity, smoking status, hypertension, drinking status, diabetes, cardiovascular disease, hypercholesterolemia, BMI and eGFR	
Carto et al. (21)		U.S.	2001–2004	*n* = 2549(0%)	hPDI	Logistical regression: OR: 0.98,95%CI0.96–0.99	–	
Liao et al. (16)		U.S.	2005–2006, 2007–2008	*n* = 2993(0%)	PID	logistic regression models: OR Q5 vs. Q1:1.43,95%CI1.01–2.03	Models adjusted for age, race, Charlson comorbidity index, alcohol consumption, smoking, BMI, and energy intake.	
Li et al. (15)	Fecal incontinence	U.S.	2005–2010	*n* = 11747(52.1%)	PID	Weighted logistic regression: OR Q4 vs. Q1:1.49,95%CI1.04–2.14	Models adjusted for sex, age, race, education level, PIR, BMI, smoking status, alcohol consumption, physical activity, hyperlipidemia, hypertension, and diabetes, CRP, and energy intake	
Zhang et al. (18)		U.S.	2009–2010	*n* = 4744(51.6%)	AID	➀Zinc:Q4 vs. Q1:OR :1.61, 95%CI1.16–2.23	Models adjusted for age, gender, race/ethnicity, levels of education, annual family income, physical activity, BMI, diabetes, hypertension, depression, serum cotinine levels, total daily energy intake, and intakes of fat, protein, calcium, sodium, and potassium	
**Analytical research (Prospective study, cohort study, case-control study)**								
**Study**	**Symptom**	**Study type**	**Country**	**study period**	**Population *n* (% female)**	**Dietary pattern**	**Outcomes**	**Observations**
Matsuo et al. (13)	Lower urinary tract symptoms	Prospective study	Japan	2014.9–2015.3	Nocturia and excessive salt intake *n* = 321(68.2%)	DASH	I:↑CLSS:0.7 ± 0.9 Δ 0.6* ± 0.9 (*P* < 0.001) C:↓CLSS:0.6 ± 0.8Δ0.9* ± 0.9 (*P* < 0.001)	–
Matsuo et al. (7)		Cohort study	Japan	2014.9–2015.3	OAB patients with excessive salt intake *n* = 98(57.1%)	DASH	I:↑OABSS:1.3 ± 1.0 Δ 1.1* ± 1.0 (*P* = 0.003) C:↔OABSS:1.3 ± 0.8Δ1.3 ± 0.8 (*P* = 0.327)	–
Loeb et al. (14)		prospective study	U.S.	1986–2016	Men with nonmetastatic prostate cancer *n* = 3505(0%)	hPDI	multivariable analysis: ➀Sexual function:Q5-Q1:−0.62 ± 0.17,95%CI −0.95 to −0.30 ➁Urinary incontinence:Q5–Q1:0.20 ± 0.14,95%CI−0.46−0.07	Models adjusted for age, time since diagnosis/primary treatment, calories, BMI, smoking, alcohol, physical activity
**Experimental study (RCT)**								
**Study**	**Symptom**	**Study period**	**Population *n* (% female)**	**Dietary pattern**	**Country**	**Outcomes**		**Observations**
Maiorina et al. (23)	Sexual dysfunction	2004–2012	Type 2 diabetes *n* = 215(50.7%)	I: Mediterranean diet C: Low-fat diet	Not mentioned	I:IIEF-5:21.9 ± 2.6Δ20.8 ± 2.2 C (low fat):IIEF-5:21.9 ± 2.1Δ19.6 ± 2.9 I:FSFI:26.3 ± 2.6Δ25.1 ± 2.8 C (low fat):FSFI:26.2 ± 2.7Δ23.9 ± 3.4		–
Esposito et al. (24)		2 years	Men with the metabolic syndrome *n* = 65(0%)	I: Mediterranean diet C: Control diet	Not mentioned	I:↑IIEF-5:18.1 ± 4Δ14.4* ± 3.8,*P* < 0.01 C:IIEF-5:14.9 ± 3.7Δ15.2 ± 3.5		–
Esposito et al. (25)		2 years	Metabolic syndrome *n* = 50(100%)	I: Mediterranean diet C: Control diet	Not mentioned	I:↑FSFI:20.1 ± 2.9▽6.4* ± 3.8,*P* < 0.01 C:FSFI:19.7 ± 3.1▽0.3 ± 0.7		–

Country: US United States of America BMI, body mass index; CLSS, the core lower urinary tract symptom score; OABSS, overactive bladder symptom score; WHR, waist-to-hip ratio; IIEF, International erectile function index questionnaire; FSFI, female sexual function index.

*, Indicates a significant value; Δ Data compared to baseline at the end of treatment; ▽ Mean changes in end-of-treatment data and baseline; ↑, Improve; ↔, unaffected; ↓, deteriorate; I, intervention group; C, control group.

### 3.1 Sexual dysfunction

Nine studies were included in the analysis of sexual dysfunction, with detailed characteristics shown in Table 2. These studies consisted of 5 cross-sectional studies and 4 prospective studies, focusing on 6 studies related to the Mediterranean diet, 1 study on pro-inflammatory diets, and 2 studies on healthy plant-based diets (hPDI). To standardize the direction of the dietary effects on sexual dysfunction, data from the pro-inflammatory diet studies were reverse-transformed to align with the overall dietary pattern effect. Except for Ruan et al.’s study, all studies assessed dietary intake using food frequency questionnaires (FFQ). For sexual dysfunction assessment, the majority of studies (except 4) used the IIEF-5 and FSFI for male and female sexual function, respectively, where higher scores indicated better sexual function. To ensure consistency across prospective studies, mean results were converted to negative values. All cross-sectional studies utilized multivariable regression analysis to control for potential confounders.

**FIGURE 2:**
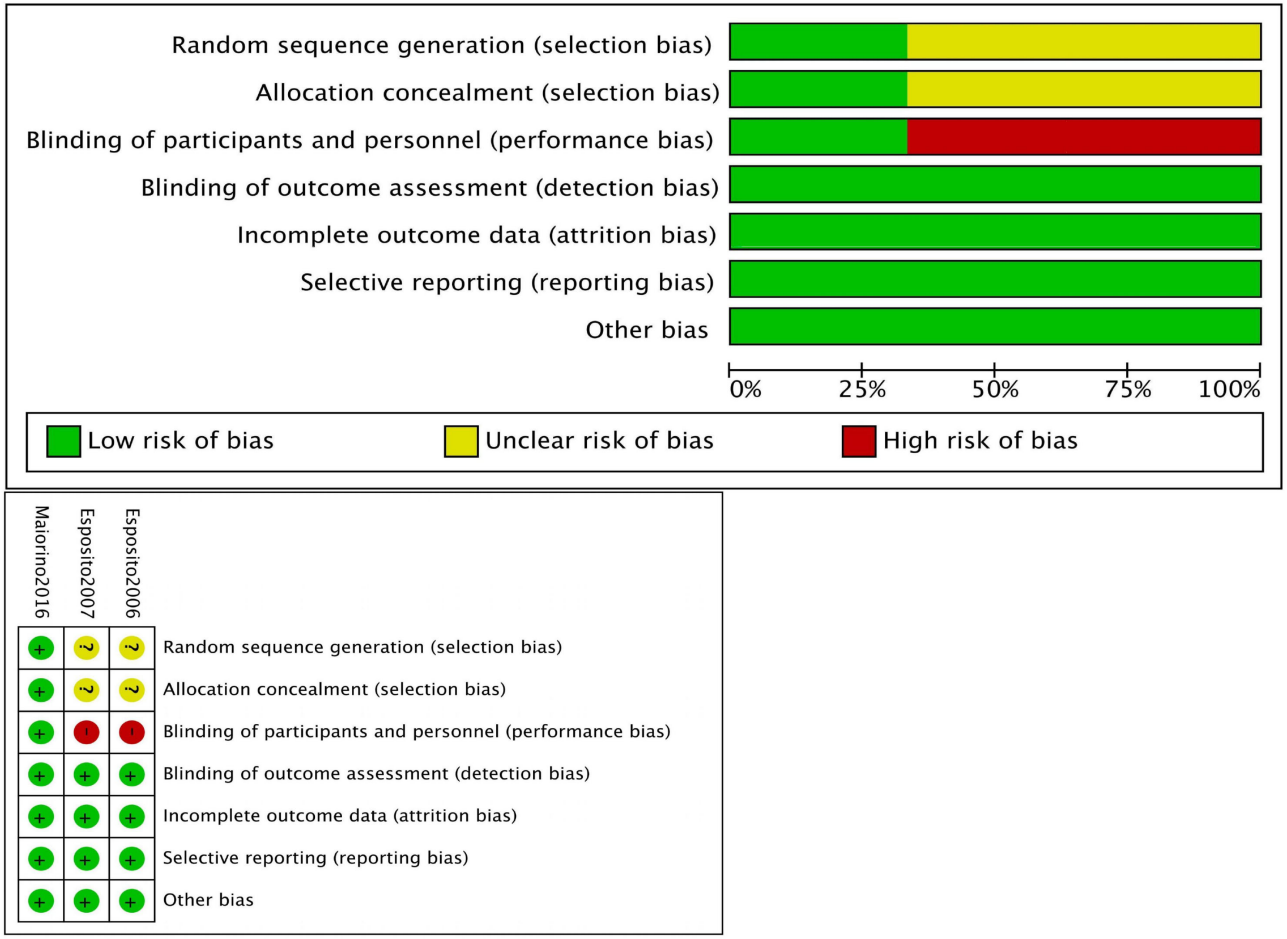
Risk of bias.

On the Newcastle-Ottawa Scale (NOS) for quality assessment, the average score of the studies was 8.1 points. Two studies achieved the maximum score of 9, eight studies scored 8, and one study scored 7, indicating high methodological quality (Figure 2).

**FIGURE 3:**
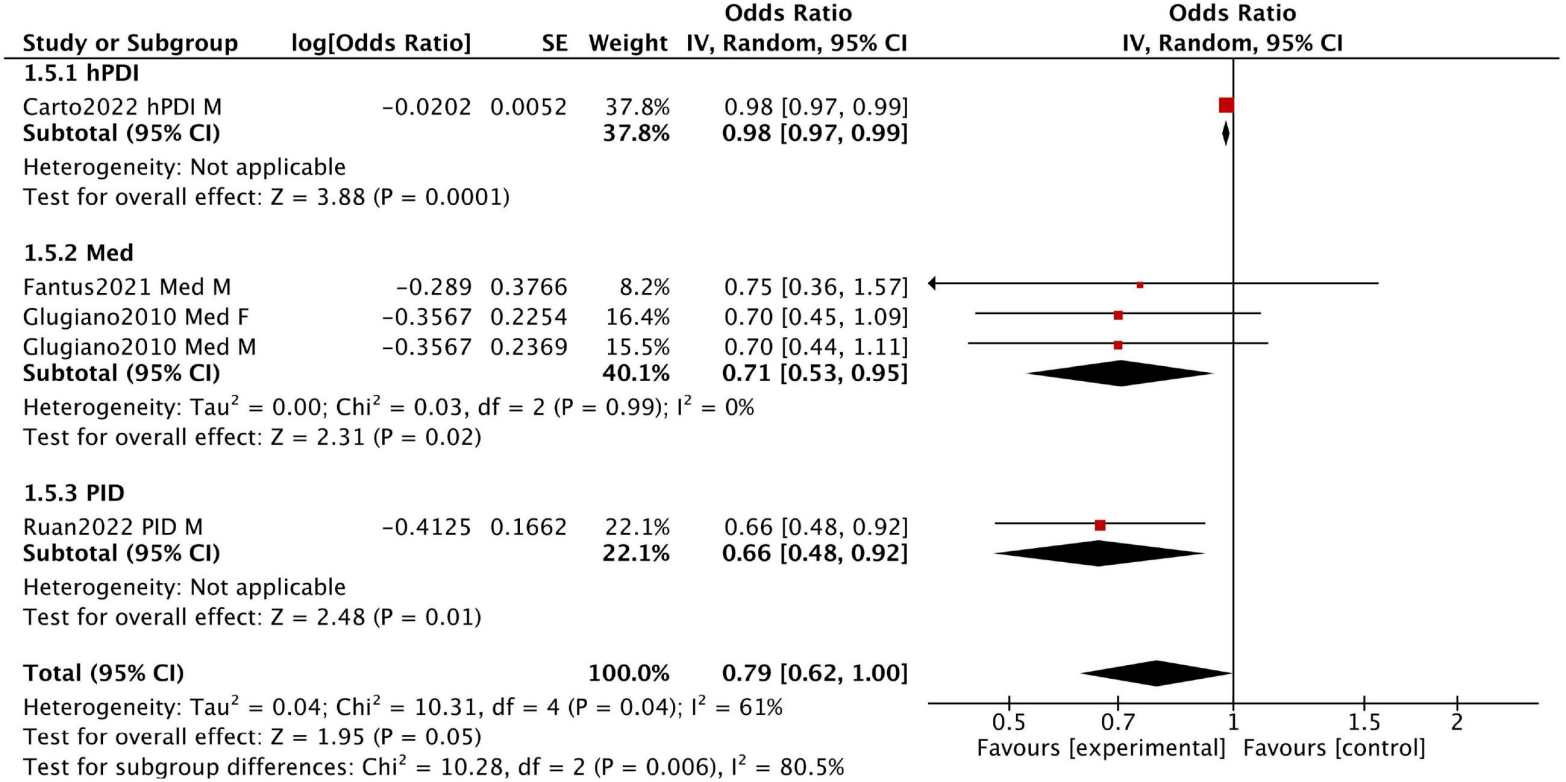
Forest plot summarizing the effect of healthy dietary patterns on sexual dysfunction based on cross sectional studies.

Figure 3 presents the results of five cross-sectional studies. With the exception of the hPDI diet, which did not show a significant improvement in sexual dysfunction, the other four studies demonstrated relatively consistent results (fixed-effect model: OR = 0.69, 95% CI [0.55, 0.85]), with low heterogeneity (I^2^ = 0%) (Supplementary Figure 1). These results indicate that the Mediterranean and pro-inflammatory diets are significantly associated with a lower risk of sexual dysfunction.

Fantus et al. analyzed data from the 2001–2004 National Health and Nutrition Examination Survey (NHANES) in the United States, including men aged 18–85 who completed the prostate and 2-day dietary questionnaires. Among 4,027 participants, 1,085 adhered to the Mediterranean diet, while 2,942 followed a non-restrictive diet. This study found no significant difference in the prevalence of erectile dysfunction between the two groups (OR 0.75, 95% CI 0.36–1.57).

Giugliano et al. conducted a survey in 2010 among men and women with type 2 diabetes in Campania, southern Italy. Participants were divided into three groups based on the Mediterranean diet score by Trichopoulou et al.: low (0–3 points), medium (4–5 points), and high (6–9 points). Men in the highest score group (*n* = 133) had a lower prevalence of erectile dysfunction than those in the lowest score group (*n* = 155) (50.3% vs. 62.6%). Similarly, women in the highest score group (*n* = 149) had a lower prevalence of sexual dysfunction compared to those in the lowest score group (*n* = 166) (48.9% vs. 57.5%).

Ruan et al. analyzed data from the 2001–2004 NHANES, including 3,693 men. The findings showed that men in the highest DII tertile had a higher risk of erectile dysfunction compared to those in the lowest DII tertile (OR = 1.12, 95% CI 1.04–1.19), suggesting that inflammatory dietary patterns may increase the risk of erectile dysfunction.

**FIGURE 4:**
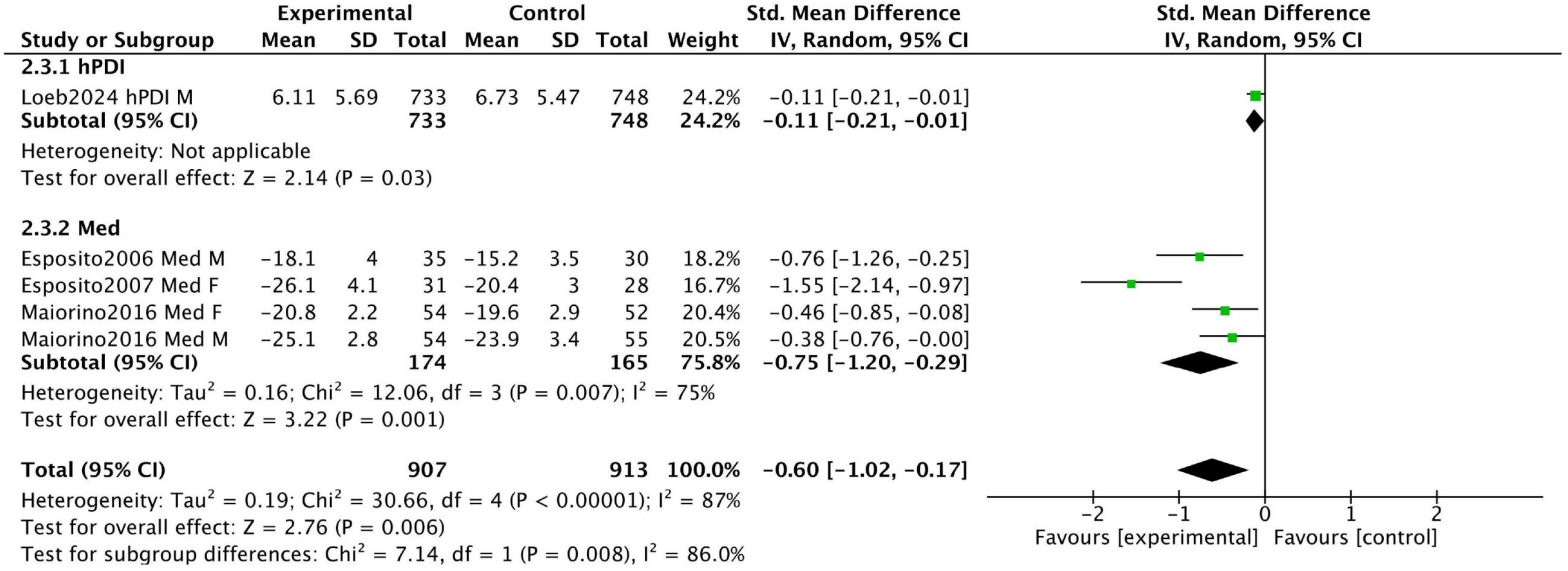
Forest plot summarizing the effect of healthy dietary patterns on sexual dysfunction based on prospective studies.

Figure 4 displays the combined results of four prospective studies, with an overall standardized mean difference (SMD) of −0.6 (95% CI [−1.02, −0.17]). This indicates greater improvements in sexual function in the intervention groups adhering to healthy dietary patterns compared to the control groups. However, substantial heterogeneity was observed (I^2^ = 87%) due to variations in participant characteristics and study design. Our sensitivity analysis revealed that the two studies by Esposito et al. (24, 25) were the primary contributors to the heterogeneity. When these two studies were removed, the analysis of the remaining studies showed a consistent, positive effect with no heterogeneity (SMD = −0.25, 95% CI [−0.49, −0.01], I^2^ = 0%) (see Supplementary Figure 2).

Esposito et al. conducted a 2-year prospective RCT in 2006 involving 65 men with metabolic syndrome. Participants were divided into a Mediterranean diet intervention group (*n* = 35) and a control group (*n* = 30). After 2 years, the Mediterranean diet group showed a significant improvement in the IIEF scores, increasing from 14.4 ± 3.8 to 18.1 ± 4, whereas the control group showed only a minimal increase from 14.9 ± 3.7 to 15.2 ± 3.5. These results suggest that the Mediterranean diet significantly improves erectile function.

In a 2007 RCT, Esposito et al. examined 59 women with metabolic syndrome, allocating 31 to a Mediterranean diet intervention group and 28 to a control group. After 2 years, the Mediterranean diet group demonstrated a significant improvement in FSFI scores, increasing from 19.7 ± 3.1 to 26.1 ± 4.1 (*P* = 0.01), while the control group showed no significant changes. These findings indicate that the Mediterranean diet significantly enhances sexual function in women with metabolic syndrome.

Loeb et al. conducted a prospective cohort study involving 3,505 participants with non-metastatic prostate cancer. The median time from prostate cancer diagnosis or treatment to the first quality-of-life assessment was 7 years. The study results indicated that a high intake of the hPDI was associated with a lower risk of sexual dysfunction. Participants in the highest hPDI quintile had a mean EPIC sexual function score of 6.11 ± 5.69, compared to 6.73 ± 5.47 in the lowest quintile (higher scores indicate worse sexual function).

Maiorino et al. conducted an 8.1-year RCT involving 215 newly diagnosed type 2 diabetes patients, randomly assigned to a Mediterranean diet group (*n* = 108) or a low-fat diet group (*n* = 107). At the end of the trial, declines in IIEF and FSFI scores were significantly smaller in the Mediterranean diet group compared to the low-fat diet group (1.22 vs. 1.18; *P* = 0.024 and *P* = 0.019, respectively). These findings indicate that the Mediterranean diet slows the deterioration of sexual function more effectively than a low-fat diet.

### 3.2 Urinary and fecal incontinence

A total of six studies on incontinence were included, comprising three cross-sectional studies and three prospective studies. The characteristics of these studies are shown in Table 2. Among these, two studies focused on the DASH diet, three on pro-inflammatory diets, and one on the healthy plant-based diet (hPDI). With the exception of the study by Li et al., all studies used urgency urinary incontinence (UUI) as the primary outcome variable. Multivariable regression analysis was employed in all cross-sectional studies to control for potential confounders and assess the relationship between dietary patterns and urinary or fecal incontinence.

**FIGURE 5:**
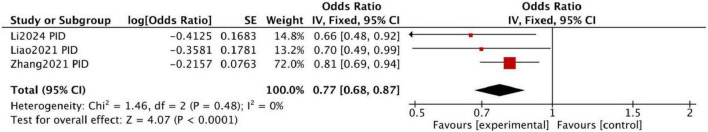
Forest plot summarizing the effect of healthy dietary patterns on incontinence based on cross sectional studies.

The results of the three cross-sectional studies are presented in Figure 5. Dietary Inflammatory Index (DII) scores were significantly associated with urinary and fecal incontinence, suggesting that reducing pro-inflammatory components in the diet may help lower the prevalence of these conditions (fixed-effect model: OR = 0.77, 95% CI [0.68, 0.87], I^2^ = 0%).

Li et al. analyzed data from 11,747 participants in the 2005–2010 NHANES database, dividing participants into quartiles based on DII scores (Q1 < 0.47; Q4 > 2.71). Participants in the highest quartile (Q4) had a significantly increased risk of fecal incontinence (FI) compared to those in the lowest quartile (Q1) (OR = 1.49, 95% CI [1.04–2.14]).

Similarly, Zhang et al. analyzed data from 3,230 women under the age of 65 in the 1999-2016 NHANES and found that women in the highest DII quartile had a significantly higher risk of UUI compared to those in the lowest quartile (OR = 1.24, 95% CI [1.07–1.44]).

Liao et al. analyzed data from 2,993 men in the NHANES dataset and found that the highest DII quintile was associated with a significantly increased risk of lower urinary tract symptoms compared to the lowest quintile (OR = 1.43, 95% CI [1.01–2.03]).

**FIGURE 6:**
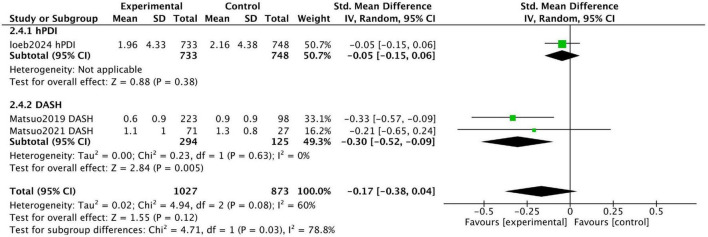
Forest plot summarizing the effect of healthy dietary patterns on Incontinence based on prospective studies.

Figure 6 displays the combined results of three prospective studies, showing an overall standardized mean difference (SMD) of −0.17 (95% CI [−0.38, 0.04]) for participants adhering to healthy dietary patterns. While there was a trend toward improvement in urinary incontinence with healthy diets, the results were not statistically significant, and substantial heterogeneity was observed among the studies (I^2^ = 60%). This moderate heterogeneity likely stems from the stark differences in the study populations and dietary interventions assessed.

Loeb et al. conducted a prospective cohort study and found no significant association between hPDI intake and urinary incontinence, with urinary incontinence scores on the Expanded Prostate Cancer Index Composite being 1.96 ± 4.33 and 2.16 ± 4.38 for the highest and lowest quintile groups, respectively (*P* = 0.14).

In contrast, two prospective studies by Matsuo et al. investigated the effect of reducing salt intake on urinary symptoms in patients with baseline high salt intake. While focusing on a single nutrient, these studies were included because sodium restriction is the cornerstone of the DASH diet, one of our target dietary patterns. Thus, these studies provide direct evidence on a key active component of this pattern. The study included patients who urinated at least once per night and had excessive salt intake (≥8 g/day for men, ≥7 g/day for women). Of the participants, 223 successfully reduced salt intake (S group), while 98 failed to reduce intake (F group). The urgency urinary incontinence score in the S group improved from 0.7 ± 0.9 to 0.6 ± 0.9 (*P* = 0.001), whereas the score in the F group worsened from 0.6 ± 0.9 to 0.9 ± 0.9. These findings indicate that reducing salt intake can improve urinary incontinence.

In a 2021 study, Matsuo et al. conducted another 12-week trial focusing on patients with overactive bladder (OAB) and excessive salt intake (≥8 g/day for men, ≥7 g/day for women). Among 98 participants, 71 successfully reduced salt intake (R group), while 27 did not (N-R group). At the end of the trial, the urgency incontinence score on the OABSS questionnaire decreased from 1.3 ± 1.0 to 1.1 ± 1.0 in the R group, with no significant changes observed in the N-R group. These findings suggest that reducing salt intake alleviates urgency urinary incontinence in patients with OAB.

## 4 Discussion

This study found that healthy dietary patterns, particularly the Mediterranean diet, are significantly associated with improved sexual function. While broadly anti-inflammatory, the Mediterranean diet’s unique composition–rich in olive oil, nuts, and polyphenols (26)–also directly improve endothelial nitric oxide production (27, 28). This dual mechanism explains why its impact on sexual health may be greater than that predicted by general anti-inflammatory indices like the DII, which are less sensitive to these specific pro-vascular, synergistic effects.

A strong explanation for the observed treatment heterogeneity emerges from our subgroup analysis of prospective studies. The pronounced treatment effect was overwhelmingly driven by trials recruiting patients with metabolic syndrome–a condition where central obesity is a cornerstone component. This patient group suffers from a confluence of risk factors, including chronic inflammation, vascular impairment, and increased intra-abdominal pressure due to excess weight. Therefore, they may be particularly responsive to dietary interventions that target all these aspects simultaneously. In contrast, Fantus et al. reported no significant association between the Mediterranean diet and erectile dysfunction, possibly due to a different definition of the diet. Their study limited daily intake to 1,800 kcal, with no more than 40% of calories from fat, omitting specific restrictions on saturated fat and cholesterol, and failing to emphasize the importance of monounsaturated and polyunsaturated fatty acids.

This principle extends beyond a single dietary model. Our analysis consistently found that other plant-rich, antioxidant-heavy dietary patterns–such as those with high Healthy Plant-Based Diet Index (hPDI) scores or increased nut and flavonoid intake–were also associated with better erectile function (29, 30). Conversely, pro-inflammatory diets as measured by Dietary Inflammatory Index (DII) (31) scores, was linked to worse PFD outcomes, particularly an increased risk of urinary and fecal incontinence. These parallel findings from opposite ends of the inflammatory spectrum reinforce the dose-dependent role of dietary inflammation in PFD pathogenesis.

While the evidence for dietary intervention in sexual dysfunction is robust, the connection to urinary symptoms is more nuanced. Although cross-sectional data show a clear link between high DII scores and incontinence, prospective evidence is less consistent. This may reflect the multiple physiological mechanisms of urinary incontinence. Obesity is one of the most significant and well-documented risk factors (32). Increased body weight raises intra-abdominal pressure, placing chronic strain on the pelvic floor muscles and bladder sphincter. Therefore, for incontinence symptoms, the benefits of healthy dietary patterns may be more strongly mediated by weight loss than for sexual dysfunction. For example, the DASH diet has been shown to aid in both weight management and blood pressure control (33). While these studies focused on sodium, the broader DASH pattern also promotes weight loss, which alleviates mechanical stress on the bladder. This dual benefit suggests that for urinary symptoms, targeted interventions addressing specific pathways and broader factors, like obesity, are both clinically valuable.

This study has limitations, including heterogeneity in dietary assessments tools, sample sizes and follow-up durations across studies. Besides, while most studies made multivariable adjustments, their observational nature limits causal inferences. A critical limitation is the potential for residual confounding, particularly from obesity. As indicated in our characteristics table, not all included studies adjusted for BMI or other adiposity measures. Therefore, it is plausible that the observed association between dietary patterns and PFD is partly mediated by changes in body weight. Future research should be designed to disentangle these effects by measuring and controlling for changes in adiposity. However, this meta-analysis provides the most comprehensive synthesis to data. We have integrated diverse evidence to demonstrate a coherent relationship between dietary patterns and PFD. Our findings provide robust, evidence-based guidance for clinicians to recommend anti-inflammatory dietary strategies, especially for patients with concurrent metabolic risk factors, to improve pelvic floor health.

## 5 Conclusion

The results provide strong evidence for the significant impact of healthy dietary patterns on pelvic floor dysfunction (PFD), particularly in improving sexual dysfunction and incontinence symptoms. The Mediterranean diet and anti-inflammatory dietary patterns are significantly associated with a reduced risk of sexual dysfunction, while the DASH diet also plays a positive role in alleviating PFD symptoms. In contrast, pro-inflammatory diets were consistently linked to a higher risk of incontinence. These results have direct clinical implications. Clinicians should consider recommending evidence-based healthy dietary patterns, such as the Mediterranean diet, as part of a comprehensive management strategy for PFD. However, further high-quality studies are required to confirm these findings and provide clearer scientific evidence to guide the development of dietary intervention strategies.

## Data Availability

The original contributions presented in this study are included in this article/Supplementary material, further inquiries can be directed to the corresponding author.

## Supplementary material

Supplementary Figure 1 Forest plot summarizing the effect of healthy dietary patterns on sexual dysfunction based on cross sectional studies (Exclude hPDI diet).

Supplementary Figure 2 Forest plot summarizing the effect of healthy dietary patterns on sexual dysfunction based on prospective studies (Exclude studies by Esposito et al).
