# Supplementary figures and images for: Healthy dietary patterns improve sexual function and incontinence symptoms: systematic review and meta-analysis of dietary patterns and dietary interventions
Source: Front Nutr. 2025 Sep 30;12:1635909. doi: 10.3389/fnut.2025.1635909 (PMC12518096; doi:10.3389/fnut.2025.1635909)

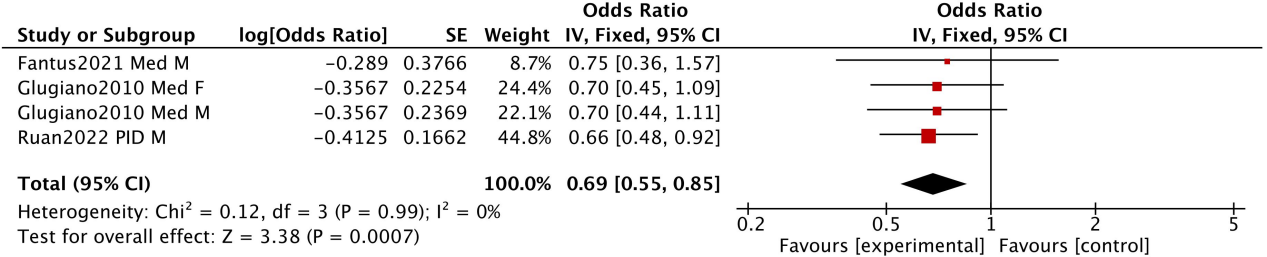

Supplement: Supplementary Figure 1 — Forest plot summarizing the effect of healthy dietary patterns on sexual dysfunction based on cross sectional studies (Exclude hPDI diet). [file Image_1.jpeg]

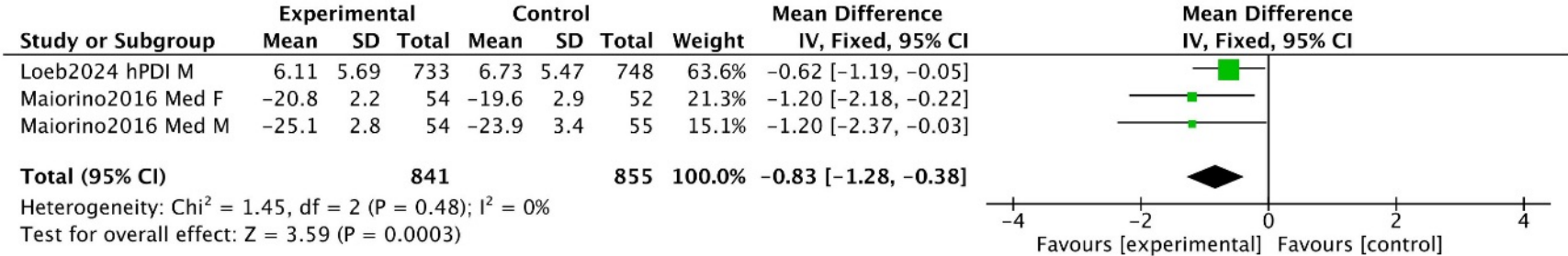

Supplement: Supplementary Figure 2 — Forest plot summarizing the effect of healthy dietary patterns on sexual dysfunction based on prospective studies (Exclude studies by Esposito et al). [file Image_2.pdf]
